# Unmet Parental Mental Health Service Needs in Neonatal Follow-Up Programs: Parent and Service Provider Perspectives †

**DOI:** 10.3390/children10071174

**Published:** 2023-07-06

**Authors:** Shayna K. Pierce, Kristin A. Reynolds, Lorna S. Jakobson, M. Florencia Ricci, Leslie E. Roos

**Affiliations:** 1 Department of Psychology, University of Manitoba, Winnipeg, MB R3T 2N2, Canada; kristin.reynolds@umanitoba.ca (K.A.R.); lorna.jakobson@umanitoba.ca (L.S.J.); leslie.roos@umanitoba.ca (L.E.R.); 2 Department of Psychiatry, University of Manitoba, Winnipeg, MB R3E 3N4, Canada; 3 Children’s Hospital Research Institute of Manitoba, Winnipeg, MB R3E 3P4, Canada; fricci@hsc.mb.ca; 4 Department of Pediatrics and Child Health, University of Manitoba, Winnipeg, MB R3A 1S1, Canada; 5 Manitoba Neonatal Follow-Up Program, Winnipeg, MB R3E 3G1, Canada

**Keywords:** mental health service use, service use barriers, parents of high-risk infants, neonatal follow-up program, service provider perspectives, mixed-methods

## Abstract

Parental mental health services in neonatal follow-up programs (NFUPs) are lacking though needed. This study aimed to determine (1) the unmet mental health needs of parents and (2) the parent and provider perspectives on barriers and opportunities to increase mental health service access. *Study 1*: Parents in a central Canadian NFUP (*N* = 49) completed a mixed-method online survey (analyzed descriptively and by content analysis) to elucidate their mental health, related service use, barriers to service use, and service preferences. *Study 2*: Virtual focus groups with NFUP service providers (*N* = 5) were run to inform service improvements (analyzed by reflexive thematic analysis). The results show that parents endorsed a 2–4 times higher prevalence of clinically significant depression (59.2%), anxiety (51.0%), and PTSD (26.5%) than the general postpartum population. Most parents were not using mental health services (55.1%) due to resource insecurity among parents (e.g., time, cost) and the organization (e.g., staffing, training, referrals). Consolidating parents’ and service providers’ perspectives revealed four opportunities for service improvements: bridging services, mental health screening, online psychoeducation, and peer support. Findings clarify how a central Canadian NFUP can address parental mental health in ways that are desired by parents and feasible for service providers.

## 1. Introduction

The Canadian Institute for Health Information reports that 14.4% of Canadian infants are born premature or with health concerns warranting admission to the Neonatal Intensive Care unit (NICU) [1]. Most parents perceive the NICU experience as a traumatic life event [2], with 18–63% of parents experiencing associated, significant mental health concerns. For example, the prevalence of depression, anxiety, and posttraumatic stress disorder (PTSD) symptomology postpartum is two to four times higher among mothers of high-risk infants [3,4,5,6] than in the general postpartum population [3,7,8]. The mental health of fathers of high-risk infants also suffers [9,10]. Research exploring this topic has been focused on the period spanning the first 14 months postpartum [11,12]. As such, there is a need to investigate the prevalence of parental mental health concerns beyond this period, particularly among parents of high-risk infants who go on to receive continued care within Neonatal Follow-up programs (NFUPs). This is important given that mental health concerns in parent’s can adversely impact parent’s adjustment to their parenting role [13,14], the parent–infant bond [15,16,17], parents’ capacity to follow complex medical regimens for their infant [18], and their infant’s development [11,19,20].

Despite the fact that 64% of NFUP parents in Canada desire mental health screening, such services are generally lacking in Canadian NFUPs [21,22,23,24], and there is no standardized national approach to addressing parental mental health [25]. Moreover, even when services are available, parents of high-risk infants can experience significant barriers to accessing them. Research involving parents whose infants are still in the NICU suggests that demographic factors (e.g., gender, marital status, the number of children within the household [12]) and factors that enable service use (e.g., education, employment status, income, time, energy, childcare, and spousal support [12,26,27]) can limit service utilization. Parents who experience high levels of trauma relating to medical concerns and procedures their infant endures while in the NICU are also less likely to utilize mental health services [28,29]. After NICU discharge, needing time to adjust to the home environment and complex medical regimens [30], experiencing feelings of vulnerability or fear, and challenges relating to scheduling appointments during service providers’ hours of operation all serve as additional barriers [27,31].

This two-part study aimed to assess parental mental health needs and elucidate how parental mental health service gaps in the NFUP program at the Rehabilitation Centre for Children (RCC) in central Canada could be improved by consolidating parents’ and service providers’ perspectives. This integrative approach is important because healthcare providers can bring unique insights into barriers to effective service delivery and because when healthcare provider and parent perspectives on barriers to parental mental health services diverge, this in itself can create barriers [31,32,33,34,35]. To meet our study goals, we utilized a convergent parallel mixed-methods design. In Study 1, we ran a mixed-methods survey online to examine parents’ experience of mental health services while they attended the NFUP across access, barriers, and service preferences. In Study 2, we conducted focus groups to explore NFUP service providers’ perceptions of barriers and opportunities to address parental mental health needs. By integrating the findings from these two studies, we sought to identify ways to help meet this parent population’s mental health needs in ways that not only consider their preferences, but that are also feasible to implement.

## 2. Materials and Methods

### 2.1. Study 1 Methods

#### 2.1.1. Participants

We recruited fifty parents of high-risk infants who attended the RCC NFUP. The sample size is consistent with that seen in other mixed-methods patient-oriented research intended to inform program development [36]. Parents who had an infant in the NFUP within the past five years were considered eligible; 85.9% of participating parents were beyond 14 months postpartum. Parents who lacked Internet access or who could not read English were considered ineligible given the studies use of an online survey in English.

#### 2.1.2. Study Procedure

Parents were recruited through the NFUP and via social media. A link to the consent form and 20 min survey housed on REDCap was emailed to interested parents. Parents who completed the survey were entered into a draw for a monetary prize.

#### 2.1.3. Materials

##### Sociodemographic Variables

Sociodemographic data collected via self-report included household size (number of adults and children), relation to the child (mother, father, other), marital status (married/common-law, divorced, separated, widowed, single/never married), employment status (full-time, part-time, on leave, not employed), household income (intervals of CAD 10,000 starting at CAD 1–10,000 and ending at CAD 140,000 or higher) and education level (ranging from some high school to PhD). Parents were also asked to supply information regarding their high-risk infant who attended the NFUP, such as the infant’s gestational age at birth and the types of services parents were involved in as part of their infant’s care.

##### Parental Mental Health

Parents in need of mental health services for depression, anxiety, and PTSD were determined using the *Patient Health Questionnaire 9-item Version* (PHQ-9) [37], *Generalized Anxiety Disorder scale* (GAD-7) [38], and the *Posttraumatic Stress Disorder Checklist for DSM-5* (PCL-5) [39], respectively. The PHQ-9 and GAD-7 assess depression and anxiety over the previous two weeks. The 9 items comprising the PHQ-9 are responded to using a 4-point Likert scale (*0 = not at all to 3 = nearly every day*), and the 7-items comprising the GAD-7 are responded to using a 4-point Likert scale (*0 = not at all sure to 3 = nearly every day*). The construct validity and internal consistency (α = 0.88–0.93) of both measures is excellent [37,40]. PTSD symptomology was assessed using the *Posttraumatic Stress Disorder Checklist for DSM-5* (PCL-5), containing 20 items responded to with a 5-point Likert scale (*0 = not at all to 4 = extremely*). The validity and internal consistency of this measure is also excellent (α = 0.90–0.95) [41,42].

Need for services was operationalized as meeting or exceeding a cut-off score of 5 on the GAD-7 and/or the PHQ-9 (signifying mild symptom severity), or a cut-off score of 33 on the PCL-5. The GAD-7 and PHQ-9 cut-offs were chosen based on score interpretation guidelines for these measures, which dictate that a conversation with a provider is warranted for those whose scores indicate mild depression or anxiety [43]. Cut-off scores for all three measures were also informed based on past work showing high specificity and sensitivity in identifying mental health service need in both general [37,38,39,42] and perinatal [44,45,46] populations.

##### Parental Mental Health Service Use and Barriers

Our team at the University of Manitoba developed a 10-item *Parent Mental Health Service Utilization* measure (see Appendix A) to assess parents’ use of mental health services and barriers and motivators to use (e.g., cost, interest, belief in need, etc.). Responses were based on parents’ use of professional, community, and self-guided mental health services, either virtually or in-person.

##### Open-Ended Questions

Open-ended questions were included within the Mental Health Service Utilization measure regarding respondents’ biggest mental health service use motivators and barriers. Additional barriers and motivators were captured by an ‘other’ option in items listing possible motivators and barriers. 

#### 2.1.4. Data Analysis

Quantitative. Data were cleaned following Meade and Craig (2012) [47] and analyzed in IBM SPSS Statistics (v.28). A sample of *N* = 49 remained, with 0 to 4.1% missing data per measure. Data were indicated as missing randomly (*p* = 0.118 to *p* = 0.634) using Little’s MCAR test. Where appropriate, expectation maximization was used to estimate missing data. A crosstab analysis examined how endorsed mental health concerns (anxiety, depression, PTSD) were associated with use of mental health services. Crosstab analyses were run to obtain chi-square values. 

Qualitative. Open-ended responses were analyzed following a conventional content analytic approach [48] to enrich our understanding of parent’s mental health service use and preferences. Responses were given an initial code that summarized the text using wording that aligned as closely as possible with parent’s wording. Next, initial codes across parent’s responses were grouped to form themes. Similarities and differences in parental experiences and preferences were captured in subthemes under each main theme along with the number of codes within each subtheme [48]. The coding process was led by the first author, who worked with two trained research assistants; consultation on coding decisions was provided by the first author’s research advisor (author 2). Rigorous and trustworthy results were ensured following Tracy’s “Big-Tent” Criteria for Excellent Qualitative Research [49]. Each coder kept an audit trail of coding challenges and resolutions reached collaboratively during weekly team meetings by using a coding journal [49,50]. Team meetings were also used to support self-reflexivity by giving the opportunity for coders to consider whether their background and beliefs would inherently bias the data analysis process. Additionally, the full coding team reviewed and refined themes after one- and two-thirds of the data were coded to ensure that emergent themes captured the depth and breadth of parents’ responses.

### 2.2. Study Two Method

RCC NFUP service providers’ perspectives on the need for parental mental health services in their program, and opportunities and barriers to address this need were assessed across two semi-structured focus groups.

#### 2.2.1. Procedure and Sample

Purposive sampling was used to recruit service providers (*N* = 5) working in the RCC’s NFUP (neonatologists, developmental pediatricians, occupational therapists, and physiotherapists). After giving informed consent, service providers completed a 5 min online demographic survey and participated in one of two 60 min focus groups on Zoom Healthcare. Discussion covered providers’ perception of the need for parental mental health services in their program, barriers and motivators to parents’ mental health service use, providers’ current approach to addressing parents’ mental health, and recommendations for service improvements. A charitable donation was provided in the NFUP’s name as honorarium. Focus groups were audio-recorded on Zoom Healthcare, transcribed and de-identified in TRINT, and analyzed in a Word document.

#### 2.2.2. Data Analysis

A reflexive thematic approach was taken to analyze transcripts from each focus group. This approach was most appropriate given that it is well aligned with an inductive and exploratory approach [51]. Coding occurred iteratively and collaboratively across six steps [52]. First, transcripts were read by the first author, and two trained research assistants gained familiarity with the data by independently reading the transcripts. Second, initial codes for each sentence within transcripts were generated independently. Third, main themes and subthemes were developed by grouping initial codes by shared meaning. Fourth, the data were reviewed by each coder to ensure that the breadth and depth of perspectives were captured within the emergent themes. Fifth, theme names and definitions were developed by reviewing content coded under each theme. Finally, results were written to relate generated themes to the original research questions. Rigorous and credible results were achieved similarly to Study 1, with the main difference being that consensus across coders was not sought. Rather, reflexivity was used to allow for nuances in data interpretation across coders [51].

### 2.3. Integration of Findings

Integration of qualitative and quantitative findings from Study 1 and Study 2 was conducted at three levels [53]. A convergent parallel mixed-methods design allowed for integration at the design level. Embedding was used to integrate data at multiple points throughout data collection and analysis (e.g., collecting and analyzing parent mixed-methods data regarding barriers to mental health service use while collecting and analyzing qualitative data from service providers on barriers and integrating findings). At the reporting level, connections between qualitative and quantitative findings from both samples were illustrated in a joint table. Additionally, the results were written using a weaving narrative approach, where parental mental health, service use and barriers, and opportunities for service development were described by consolidating qualitative and quantitative data from both samples [53].

## 3. Results

### 3.1. Participant Characteristics

#### Parent and Service Provider Demographics

A summary of parent and service provider demographics can be found in Table 1 and Table 2, respectively.

### 3.2. Integrated Perspectives of Parents and Service Providers

To find answers to our four main research questions, data regarding parents’ and service providers’ perspectives were consolidated as outlined in Table 3. In presenting the findings relating to each question below, we refer to relevant themes and subthemes that were identified in service providers’ perspectives through the analysis of the data gathered in Study 2. This qualitative analysis was completed prior to data consolidation.

#### 3.2.1. Parental Mental Health in the Neonatal Follow-Up Program

Four subthemes concerning parental mental health were developed from provider focus groups: (1) parental mental health concerns, (2) risk factors for worsening mental health, (3) protective factors against worsening parental mental health, (4) and parents’ coping styles. Qualitative and quantitative data were woven into the discussion of each subtheme to consolidate perspectives across the parent and service provider samples.

**Parental Mental Health Concerns**. Service providers felt that NFUP parents were at risk for multiple mental health concerns, which was supported by quantitative findings in the parent survey. Of the full sample of parents, 13 (26.5%) endorsed no mental health concerns, 15 (30.6%) endorsed one mental health concern, 11 (22.4%) endorsed two mental health concerns, and 10 (20.4%) endorsed three mental health concerns. The comorbidity of depression and anxiety was found to be higher (40.8%) than that for either depression and PTSD (22.4%) or anxiety and PTSD (20.4%).

*PTSD.* Service providers explained that most NFUP parents experience posttraumatic stress from their time in the NICU, with one service provider pointing out that “*their baby can die at any time”* (SP2). Service providers’ perception was supported by parents’ responses on the PCL-5, which indicated that 13 (26.5%) endorsed posttraumatic stress levels warranting mental health service use. The NICU was the most common trauma context identified by parents, with one parent writing “*our twins were born at 23 weeks, and one was in the NICU for six months. Our firstborn only lived three weeks. A lot of hard things happened in that time, to our son and the relationship between my husband and I”* (ID 36).

*Anxiety.* Service providers noted their perception that parents attending the NFUP present as anxious. Total scores on the GAD-7 showed that twenty-five parents (51%) endorsed anxiety levels that warranted mental health service use, with eleven (22.4%) endorsing mild anxiety, seven (14.3%) endorsing moderate anxiety, and seven (14.3%) endorsing severe anxiety. The most discussed content of worry among parents was their infant’s prognosis. One parent wrote “*Are her lungs going to be alright? Will [bronchopulmonary dysplasia] limit her lifespan? How to keep her safe from illness? Will she ‘catch up’ developmentally? Will she always be so tiny? Will she develop speech well? (Yes) Will she resent that we picked Audio-Verbal therapy rather than sign language as a speech acquisition modality? Is she eating enough? Is isolating against [respiratory syncytial virus] and then COVID-19 going to have put her behind socially? Are other children going to tease her for her small stature? Will she have friends? Will she be ready to start kindergarten at 5?”* (ID 79). One provider explained that beyond worrying about their infant’s prognosis, parents are worried they “*won’t be able to cope, won’t be able to meet baby’s needs and that their child’s outcomes are a reflection of them as a parent”* (SP1). Further, parents worried about their interpersonal relationships (e.g., partner and other family members), resources (e.g., finances, childcare for other children), their ability to manage role demands (e.g., homeownership, work, large to-do lists, caring for other children), doing enough for their children, and something bad happening to them or their children.

*Depression.* Service providers explained that they had limited experience identifying depression among adults, but they characterized parents whom they perceived as withdrawn and presenting with low mood during appointments as having depression. This perception was supported by parents’ responses on the PHQ-9, which showed that twenty-nine (59.2%) parents endorsed levels of depression that warrant mental health service use, with seventeen (34.7%) endorsing mild depression, four (8.2%) endorsing moderate depression, four (8.2%) endorsing moderately severe depression, and four (8.2%) endorsing severe depression. One parent wrote “*I fell into a deep depression during my pregnancy with my twins. Not able to get out of bed, couldn’t stop crying, wasn’t able to care for my then 3-year-old”* (ID 102).

**Risk Factors to Worsening Parental Mental Health**. Service providers discussed their perception of risk factors for worsening parental mental health, elucidating three subthemes: (a) transitions, (b) managing multiple demands, and (c) social disparities.

*Transitions.* All providers discussed three key transition timepoints where parents are at risk of worsening mental health: leaving the NICU, entering the NFUP, and the end of maternity leave. Transitions were characterized as periods with gaps in mental health support and increased parental stress resulting from receiving less reassurance from medical professionals and experiencing uncertainty around their infant’s prognosis and their ability to care for their infant. One service provider explained: “*Parents go from having constant healthcare support and data to support that their infant is okay [in NICU] to being on their own”* (SP3). Parents identified a fourth transition as a risk factor for worsening mental health, namely the transition into being pregnant again following traumatic birth experiences that resulted in their baby being admitted to NICU. One parent captured this, writing “*After my first child was born prematurely and had an extended NICU stay, I was concerned for my 2nd child’s health”* (ID 47).

*Managing Multiple Demands.* Service providers explained that, along with the typical demands of caring for a newborn, parents attending the NFUP must attend multiple healthcare appointments and learn and perform treatment regimens for their infant. Indeed, parents in this sample were attending an average of two services for their high-risk infant (*M* = 2.0, *SD* = 2.2), with 20.4% having accessed five services for their infant in the past. Service providers also discussed additional demands that parents manage, including caring for other children or family members, which can lead to exhaustion and worsening mental health. One parent echoed this, saying “*I’m concerned I am not doing enough for my children. They both need a great deal of therapy. There is never enough time in the day to do it all. I feel like I am failing them both”* (ID 47). This can be a particular concern for single parents (who comprised 12.2% of the sample) who must manage multiple responsibilities without the support of a partner. Further, service providers perceived some parents as struggling or feeling guilty around directing more attention and providing more care for their high-risk infant than their other, typically older, children. This is notable, as 67.3% of parents in the present sample identified as having multiple children. 

*Social Disparities*. Beyond interpersonal relationships, social disparities were perceived by service providers as a risk factor for worsening parental mental health. Service providers mentioned that parents of lower socioeconomic status tend to have limited resources, putting them at greater risk of mental health concerns. One service provider stated “*Generally, families with more resources may still have as much anxiety, but they’re better able to cope with that. It can be more resilient than families who have other stressors in addition to what they have”* (SP1). This is concerning as 20.3% of families identified as low income (CAD 0–40,000).

**Protective Factors Against Worsening Parental Mental Health.** Two subthemes emerged relating to service providers’ perception of factors protecting parents from worsening mental health: (1) social support, and (2) support within the healthcare system.

*Social Support*. All service providers noted social support (family, friends, and community) as “the most important” protective factor against worsening parental mental health. Service providers perceived parents who had more social support as coping better with the stress of caring for their high-risk infant than those with less or no social support. One service provider stated, “*For sure, the extra family support or friend support is huge, even for just attending appointments, someone else watching the other kids or being an extra support person at the appointment”* (SP4). Most parents (51.0%) felt that having a child in the NFUP did not change how supported they felt by their social network, and 26.5% of parents said that it made them feel more supported. Moreover, many parents felt that having an infant in the NFUP either did not change (38.8%) or improved (38.8%) how supported they felt by their partner.

*Support from the Healthcare System*. Beyond interpersonal support, a few service providers expressed the view that parents who felt supported and connected to the healthcare system tended to be more optimistic and engaged than parents who did not. Service providers defined connectedness to the healthcare system as parents having regular access to services and providers (e.g., family physicians or social workers). However, they noted that healthcare connectedness is often not possible for families, in part because services are overwhelmed.

**Parental Coping Styles**. All service providers discussed seeing a range of mental health coping strategies among parents who have a high-risk infant attending the NFUP. Based on their descriptions, three parent coping styles were identified: seekers, attenders, and avoiders. “Seekers” actively seek out education and support resources for their child and themselves beyond that which is offered while in the NFUP. One service provider captured this by explaining how some parents “*really seek out a lot of resources, they love checklists and are on it the whole time, their whole day is therapy, enrichment, all that”* (SP3). “Attenders” participate in what is offered but do not seek extra resources or support. Finally, “avoiders” cope with their mental health while in the NFUP through not participating or seeking available resources. For example, when describing how some parents become overwhelmed by hearing about their infant’s health in NFUP appointments, one service provider explained that “*attending appointments for them might be hard because they have to face all those things”* (SP3).

#### 3.2.2. Current Approach to Addressing Parental Mental Health and Parents’ Service Use

Service providers were asked to describe their current approach to addressing parents’ mental health in the NFUP. This discussion revealed two subthemes: (1) there is no process or person in place, and (2) where do we refer parents? After describing these themes, we present information about parents’ self-reported mental health service use.

**There is no Process or Person in Place**. All service providers discussed that, unfortunately, there is no parental mental health screening tool or process within their program. Their current approach involved briefly asking parents how they are doing during appointments and only discussing mental health further with parents who express that they are not doing well—which, they noted, rarely occurs as parents are often focused on absorbing the information related to their child during the appointment. One service provider explained “*We’re very likely not exploring it to the need that it needs to be explored for the family. If the family gives a hint, then definitely, we’ll explore. We’ll ask how things are. And when you come to see a physician or maybe a therapist, you may say fine. Right? And it’s not fine. But if the family doesn’t open up we may not have the time ability to fall in this as you would if you had somebody as part of the program who could contact them and say how things are going and discuss”* (SP1). Another service provider expressed how they “*sigh with relief”* (SP5) when parents respond to their mental health check-in by saying that they are doing fine because they are unsure of how to address parental mental health; this was met with agreement by the other service providers.

**Where do we Refer Parents?** Service providers discussed a lack of options for referrals to address parental mental health. They noted that, unlike many other NFUPs in the country, no social worker is connected to their NFUP due to staff shortages and heavy caseloads. They described referring some families to community programs that incorporate addressing parent mental health into their programming, such as the Families First program, but added that these programs have lengthy waitlists. One provider said, “*Even for families where the children have lots of needs, medical needs, developmental needs, you do make referrals to those supports, but they sit on a waitlist for ages* (SP1).” Service providers also mentioned that they do provide parents with brochures explaining mental health programs that offer sliding scales. Although parental mental health programs do exist and are needed by parents, less than 50% of this sample of parents reported using a mental health service. 

**NFUP Parents’ Current Mental Health Service Use**. At least one mental health service was accessed by 22 parents (44.9% of the sample). Interestingly, four of these individuals did not endorse symptoms deemed to warrant service use (i.e., mild-to-moderate anxiety/depression or clinically significant PTSD), suggesting that they had either responded well to services provided or were seeking services for conditions that were not considered in the present investigation (e.g., stress). Among the service users, roughly equal numbers accessed formal services (*n* = 8), informal services (*n* = 6), or a combination of both (*n* = 8), overall; however, the latter option was preferred by most service users who reported more severe symptoms, including 7 of 13 service users endorsing co-morbid mental health conditions. Individual therapy was the most accessed form of formal mental health service (reported by 28.6% of the sample), and informal mental health services were most accessed online (reported by 22.4% of the sample).

A total of 27 parents (55.1% of the sample) reported that they did *not* access mental health services; this subgroup included 80% of all fathers (*n* = 4) and 52% of all mothers (*n* = 23). Of great concern, it also included 50% of those endorsing symptoms deemed to warrant service use (*n* = 18). Although many of these individuals endorsed mild symptoms of anxiety or depression, several endorsed more serious concerns. Indeed, over a third of those endorsing co-morbid mild-to-severe mental health conditions (*n* = 8) and approximately one quarter of those endorsing clinically significant PTSD (*n* = 3) did not access mental health services.

Expected cell sizes were too small to break down mental health service use for anxiety and depression by symptom severity. However, overall, there was no association between whether a parent endorsed symptoms of mild-to-severe depression ($\chi^{2}\left(1\right)=1.34,\;p=0.247$) or anxiety ($\chi^{2}\left(1\right)=1.63,\;p=0.201$) and whether they used a mental health service. In contrast, here was a significant association between whether a parent endorsed clinically significant symptoms of PTSD and whether they used a mental health service ($\chi^{2}\left(1\right)=7.34,\;p=0.007$). The odds of parents using mental health services were 7.54 times higher if they endorsed PTSD symptoms than if they did not.

#### 3.2.3. Barriers to Parents’ Mental Health Service Use

Both parents and service providers discussed their perception of barriers to parental mental health service use within the NFUP. The service providers’ discussion regarding the barriers in their program revealed two subthemes: (1) parent and (2) organizational barriers. Such perceptions were based on observations made during their lived experience working with parents in the program.

**Parent Barriers**. Service providers discussed three perceived barriers to parents using mental health services relating to parents’ (a) readiness, (b) attitudes and beliefs about mental health, and (c) reduced capacity for service involvement.

*Readiness.* All service providers discussed that a parent’s lack of readiness for accessing mental health services was a barrier to their mental health service use. Service providers explained that parents are so focused on caring for and meeting the complex demands of their infant that they are often not ready to focus energy on themselves.

*Attitudes and Beliefs About Mental Health.* Service providers and parents identified stigma as a barrier to parental mental health service use, with one parent saying they were “a*fraid of friends and colleagues knowing”* (ID 30). Moreover, service providers discussed that being unable to recognize or communicate when they were struggling with their mental health was a barrier for some parents. Many parents identified uncertainty around how to access mental health services (18.4%) and how it would help them (10.2%) as barriers to them seeking help.

*Reduced Capacity for Service Involvement.* Service providers felt that resource insecurity reduced parents’ capacity for service involvement. Key resources identified by service providers as necessary for parents to participate in mental health services included the parent having time, finances, childcare, and transportation to services. Parents reinforced this. Thus, not having the time or energy was identified as the biggest barrier to parents accessing mental health services (38.8%), followed by cost (18.4%). Not having the time or energy was also connected to prioritizing the child by a few parents, with one saying “*Just not wanting to put in the effort to take the time for myself to go and do it even though I know I feel better after. Always putting myself and my needs at the bottom of the barrel”* (ID 102). Childcare and transportation were also identified by parents as resources needed to attend mental health services.

**Organizational Barriers.** Service providers also talked about organizational barriers that prevent them from being able to connect parents to mental health services. These included (a) providers’ lack of training in adult mental health, (b) appointment time and demands, (c) a lack of mental health resources, and (d) the program’s structure of care.

*Provider’s Lack of Training.* All service providers discussed that they lack training in adult mental health, which they perceived as a barrier to parents being able to use mental health services. They all expressed a desire for training in how to recognize common mental health concerns among parents, when to connect parents to mental health supports, and where to refer them. One service provider stated, “*I always think that there’s more I can do, and I don’t know how”* (SP5).

*Appointment Time and Demands.* All service providers explained that even if they knew how to recognize and address parental mental health, they are limited by the one-hour length of the assessment appointments. One service provider explained “*We have an appointment that is one hour long. And during the appointment, I feel that sometimes I have to do so many things because this is well, doing all the questions about health issues, examining the baby, talking to their families, doing trying to assess and make a diagnosis, doing their referrals, and then because we are part of research projects, taking their consent for research. And so, by the end of the appointment, it’s like the hour is gone”* (SP5). COVID-19 presented nuanced challenges that made virtual appointments feel even shorter, such as internet connection issues and distractions in the home (e.g., simultaneously caring for other children).

*Lack of Mental Health Resources.* All service providers noted that they lacked staff within the program with the time and training to address parental mental health (e.g., a nurse, psychologist, or social worker), funding to integrate a mental health professional into the NFUP, knowledge of referral options, and a referral process. Service providers described the difficulty of having to rely on external agencies to support parents’ mental health as it required them to communicate between agencies and follow-up. This meant that families often ended up stuck on waitlists. One service provider captured this, saying “*I mean, we can refer out to other programs. There’s a few things out there. But they’ve been limited with COVID too, and they have their own restrictions or some barriers to accessing some of those also”* (SP4). 

*NFUP Structure of Care.* Service providers noted that they perceived the disconnect between the NICU and the NFUP as hindering parents’ mental health service use and continuity of care. One provider explained “*We are separate from the NICU, a separate location, different separate program. So, we don’t have knowledge of the support that they had in NICU, nor can we refer them back to the social worker who was involved with them in NICU. So, we don’t know their trajectory and what might be available”* (SP1).

#### 3.2.4. Informing the Development of Parental Mental Health Services in the NFUP

Service providers and parents provided insights that informed the development of parental mental health services or the adaptation of current services to address parents’ unmet mental health needs. Service providers’ discussion related to this topic revealed four subthemes: (1) service modality preferences, (2) bridging the gap between NICUs and NFUPs, (3) parent support groups, and (4) psychoeducation for service providers and parents.

**Service Modality Preferences.** Based on virtual appointment attendance during COVID-19, service providers suggested that it may be easier for parents to attend mental health services virtually. However, they also expressed a desire for integrated in-person mental health services that parents could attend while their infant was being seen in the NFUP. Most parents (71.7%) supported the use of integrated mental health services, with 65.3% saying that they would prefer to attend in-person. Only 22.4% of parents expressed that they prefer to access mental health services via telephone, and only 12.2% preferred video links. Parents’ most preferred informal service was online mental health information, and their most preferred formal service was individual therapy.

**Bridge the gap between NICU and NFUP**. Service providers discussed bridging the gap between the NICU and NFUP to address organizational barriers to parental mental health services in NFUPs. They all supported the idea of bridging the gap through the development of a mental health screening program that begins at NICU discharge and connects parents to supporting resources while waiting to begin the NFUP and during the NFUP. Having a professional who specializes in adult mental health perform parental mental health screening at NICU discharge was highlighted as important for early intervention by all service providers. The mental health professional was seen as “*a link between the NICU program and the NFUP where they help with that communication and warning us like this is one we’re aware of, they might have trouble with attending. This is the support we’re providing. You can call me if you need help with your appointments, that kind of thing”* (SP4). Further, all service providers felt that stigma around accessing mental health supports could be mitigated by making mental health screening standard for all parents being discharged from NICU and accessing NFUP.

**Parent Support Groups**. All service providers discussed how peer-led peer support groups at major transitions may mitigate the risk of transition periods worsening parental mental health. One service provider suggested developing a peer mentorship program where parents waiting to begin the NFUP are paired with parents already attending the NFUP. Such partnerships would allow new parents to access social support early and mitigate uncertainties regarding entering the NFUP by having someone they can contact to share experiences and answer questions.

**Psychoeducation for Service Providers and Parents.** All service providers discussed wanting psychoeducation resources for themselves and to provide to parents. Desired resources included brochures and books explaining common mental health concerns, how to identify them, and resources to address them. Service providers also wanted additional training regarding parental mental health concerns, with one stating “*if we are going to spend more time exploring mental health, then some staff development on the best way to do that is needed. You know, the benefits, the limitations, some parameters”* (SP1).

## 4. Discussion

This two-part study aimed to elucidate how parental mental health and related service gaps in a the NFUPs can be addressed by consolidating parents’ needs, barriers, and service preferences with service providers’ perspectives on barriers and feasible service improvements. The integrated findings elucidated provincial and national opportunities to address parental mental health in NFUPs that support parent engagement and are feasible for service providers. Such opportunities include bridging the service gap between NICUs and NFUPs, and developing an online platform that offers psychoeducation and peer support for both parents and providers. Identified opportunities and barriers to their successful use and implementation will help mitigate risk that poor parental mental health poses to the parent, infant, and family at large.

### 4.1. Parental Mental Health in the NFUP

Our findings support the view that parents of children in an NFUP are at elevated risk for mental health concerns. Indeed, (a) the proportion of parents who were experiencing a mental health concern warranting the use of a service was almost twice as high as that seen in parents of non-high-risk infants [54]; (b) symptoms of depression, anxiety, and PTSD were reported at rates more than double those previously reported for non-NFUP [8] or general [3,7,55,56] postpartum samples; and (c) the proportion of parents experiencing multiple mental health concerns was triple that reported in general postpartum samples [57]. These results extend previous studies by illustrating that parents of high-risk infants remain at an elevated risk of mental health concerns beyond one year postpartum.

### 4.2. NFUP Parents’ Mental Health Service Use

Previous research has highlighted NICU staff members’ perception that parents need to be offered mental health services upon NICU discharge [58]. The current study extends this by showing that these needs remain unmet in NFUPs. Thus, half of the parents who met criteria for at least one mental health concern were not accessing a mental health service, despite an expressed interest in doing so. This suggests a need to improve mental health literacy among parents and service providers, and to incorporate parental mental health screening—particularly at each key transition point in a family’s healthcare journey (e.g., NICU discharge to home, home to NFUP, NFUP discharge). Having an integrated mental health professional on staff could help to ensure that parents are matched to appropriate services in a timely fashion, with those identified as having mild concerns being encouraged to access informal services and those with moderate or severe concerns being encouraged to seek formal services. This approach could go some way to addressing the extremely long waitlists for formal services that parents experience [25].

### 4.3. Barriers to Parental Mental Health Service Use

The current findings extend earlier claims based on samples of service providers and of NICUs and general postpartum mothers that resource insecurity (time, energy, cost, childcare, and transportation if services were in person) [12,27,59,60], low mental health literacy (e.g., lacking an understanding of when mental health support is needed) [4,27], and institutional factors (e.g., disconnection between NICUs and NFUPs, limited resources, time within appointments to address parental mental health, and referral options) [31,34,35] seriously impact parental mental health service use. The fact that service providers also expressed their lack of training in adult mental health as a key barrier even though they had an average of 8.3 years of experience working within the program appears at odds with a previous study showing that NICU providers’ competence and years of experience are positively correlated [58]. However, service providers’ discussion of individual differences in parental mental health coping styles may provide some new insights into why parents’ and healthcare providers’ perspectives on barriers to service use are not always congruent [31]. The current results suggest that, whereas fear of bad news regarding their infants’ prognosis may be a barrier for avoiders, seekers and attenders may be unaffected or, indeed, motivated by fear.

### 4.4. Informing the Development of Parental Mental Health Services in the NFUP

Parents expressed a preference for services to be delivered in-person or by telephone. Parents also expressed interest in couples or family therapy and wellbeing apps. Parents and service providers also identified strategies to mitigate parent and organizational barriers to address parents’ unmet mental health support needs. As noted earlier (see Section 4.2), service providers expressed the need for a trained professional (e.g., a social worker) to be integrated into multidisciplinary NFUP teams to help bridge the gap between NICUs and NFUPs, connect parents to mental health supports as needed, and follow up with families. The development of a similar program is supported by the extant literature, with one review noting that screening and support for parental mental health at NICU discharge are absent and needed [61].

Additionally, service providers suggested the development of peer support groups to mitigate worsening parental mental health during key transition points (e.g., NICU discharge, waiting for NFUP, end of maternity leave). An online (website, forum, app, podcast) peer support and psychoeducation resource provided nationally may help to address parental mental health across Canadian NFUPs. Findings from the current study serve as preliminary evidence that such a platform would be desired by parents’ and would mitigate the biggest identified barrier to receiving in-person support—resource insecurity. Parents continuing to NFUPs could be provided access to secure websites when discharged from the NICU. Within this website, organizing peer support forums by child age and diagnosis could provide parents with continued support over their child’s life. Moreover, families who have a child with a rare condition could be supported by ensuring that a developed website is available nationally to foster interprovincial support. Previous literature supports the effectiveness of online platforms offering both peer support and psychoeducation in improving mental health outcomes among NICU parents over online resources only offering psychoeducation [62].

### 4.5. Limitations

This study focused on and recruited from one NFUP. As such, the generalizability of the findings to other jurisdictions (Canada and internationally) is unknown, warranting future research. Chi-square analyses could not be conducted to determine whether there were differences in the demographics of those who did not vs. did use a mental health service because expected cell sizes from Study 1 were too small. While this was not a key objective within the present study, it may give further insight into barriers and facilitators to this parent population using certain types of mental health services. Conducting a multi-center study would allow for the recruitment of larger parent samples.

Sample size was also an issue in Study 2, and this may limit the generalizability of the findings. However, qualitative research methods, such as focus groups, often prioritize in-depth exploration over generalizability. Nonetheless, although the service providers who took part in Study 2 provided valuable qualitative data for informing service improvements, we recognize that each service provider’s perspective cannot be considered representative of other providers from their field. As such, the findings of Study 2 should be considered preliminary. Future researchers should aim to recruit larger and more diverse samples, including NFUP nurses (who play a critical role within NFUPs) as well as hospital administrators and other stakeholders not included in the present investigation, who may provide unique insights that will help to inform a national approach to addressing parental mental health in NFUPs.

### 4.6. Conclusions and Future Directions

The findings from the current study add to the literature suggesting that, despite the high prevalence of mental health concerns among NFUP parents, NFUPs lack internal and external mental health supports for parents. By consolidating parents’ and service providers’ perspectives, we identified feasible opportunities to address these unmet needs.

Future research should seek to understand non-birthing parents’ mental health service needs and preferences in NFUPs to ensure that nuances in their mental health support needs are met. Research comparing whether parents’ mental health support needs and preferences differ based on their infant’s condition would inform ongoing international efforts for mental health screening in NFUPs. Finally, future research should include multiple Canadian NFUPs to expand upon the developed considerations and improve generalizability. Doing so would serve to inform a national standard of care for parental mental health in NFUPs. 

## Figures and Tables

**Table 1:** Parent Sociodemographic Information (*N* = 49).

Sociodemographic Variable		Mean and *SD* or *n* (Frequency)
Age		*M* = 32.5 years, *SD* = 6.8
Gender	Female	44 (89.8%)
	Male	5 (10.2%)
Marital Status	Married/Common Law	43 (87.8%)
	Single	6 (12.2%)
Number of Children		*M* = 2.1, *SD* = 1.1
Age of Child Accessing Services		*M* = 2.3 years, *SD* = 1.4
Race/Ethnicity	Indigenous Origins	7 (16.7%)
	European Origins	23 (54.8%)
	Other	11 (28.5%)
Education	High school	12 (24.5%)
	College/Technical	17 (34.7%)
	Bachelors	14 (28.6%)
	Masters or Professional Degree	6 (12.2%)
Employment Status	Full-time	18 (36.7%)
	Part-time	11 (22.4%)
	Not Working	20 (40.8%)
Income	CAD 0–40,000	10 (20.4%)
	CAD 40,001–80,000	12 (24.5%)
	CAD 80,001–120,000	13 (26.5%)
	CAD 120,000+	14 (28.6%)

**Table 2:** Service Provider Sociodemographic Information (*N* = 5).

Sociodemographic Variable		Mean and *SD* or *n* (Frequency)
Age		*M* = 50.4, *SD* = 13.6
Gender	Female	5 (100%)
Race/Ethnicity	European Origins	3 (60%)
	Latin, Central, and South American Origins	2 (40%)
Education	Bachelor’s degree	1 (20%)
	Professional degree	3 (60%)
	PhD	1 (20%)
Occupation	Developmental Pediatrician	2 (40%)
	Neonatologist	1 (20%)
	Occupational Therapist	1 (20%)
	Physiotherapist	1 (20%)
Years Working in Field		*M* = 22.8, *SD* = 9.7
	0–10	1 (20%)
	11–20	2 (40%)
	31–40	2 (40%)
Employment Status	Full-time	5 (100%)
Years Working in the Neonatal Follow-up Program		*M* = 8.3, *SD* = 5.8
	0–10	3 (60%)
	11–20	2 (40%)

**Table 3:** Summary of Data Consolidation Across Study 1 and Study 2.

Research Question	Consolidated Data	
	Parents	Service Providers
1. To what extent are there unmet parental mental health support needs in this central Canadian NFUP?	Anxiety, depression, and PTSD scores on the GAD-7, PHQ-9, and PCL-5 respectively, and demographics	Service providers’ perspectives on parent mental health, risk and protective factors, and coping styles
2. How are parents and service providers currently addressing parental mental health?	Parents’ current and past mental health service use from the mental health service use measure	Service providers’ current process to addressing parental mental health in NFUPs
3. What are parents’ barriers to accessing mental health services and service providers’ barriers to implementing them?	Barriers identified within the mental health service use measure	Identification of organizational barriers across focus groups
4. What opportunities do parents and service providers see to address parental mental health in NFUPs?	Desired types and modalities of mental health services identified in the mental health service use measure	Identification across focus groups of what opportunities can feasibly be developed and implemented

## Data Availability

The data presented in this study are available on request from the corresponding author. The data are not publicly available to protect the privacy of participants, in accordance with the Fort Gary Campus Research Ethics Board of the University of Manitoba.

## Appendix A

Parent Mental Health Service Utilization Questionnaire

1. The following are a number of mental health services that you may have accessed. Please check the box for each service you have accessed in the past year.
  ∘ Individual counselling/therapy
    ▪ In-person
    ▪ Virtually
  ∘ Group-based counselling/therapy
    ▪ In-person
    ▪ Virtually
  ∘ Couples Counselling/therapy
    ▪ In-person
    ▪ Virtually
  ∘ Family counselling/therapy
    ▪ In-person
    ▪ Virtually
  ∘ e-Mental Health services (e.g., online therapist-guided self-help, online chat support, online peer- or therapist-coaching)
  ∘ Mental health crisis line (e.g., Crisis Services Canada)
  ∘ Seeking mental health information online
  ∘ Well-being phone apps (e.g., guided meditation)
  ∘ Other: _______________
2a. If you have NOT accessed any mental health services, please indicate the reasons why you have not accessed mental health services (select all that apply):
  ∘ Too costly
  ∘ Do not believe it would help
  ∘ Unsure of how to access mental health services
  ∘ Do not have time or energy
  ∘ Not interested in seeking services
  ∘ Do not believe I need services
  ∘ Decline to Respond
  ∘ Other: _______
  **The following item will appear as a sliding scale (continuous variable)*
2b. To what extent do you find each of the following to be a barrier to you using mental health services?
  ∘ Too costly
  ∘ Do not believe it would help
  ∘ Unsure of how to access mental health services
  ∘ Do not have time or energy
  ∘ Not interested in seeking services
  ∘ Do not believe I need services
3. Describe some of the things that are getting in the way of you accessing mental health services.
4. What’s the biggest barrier of you accessing mental health services?
5. Describe some things that might make it easier for you to take care of your mental health or access services:
6. If you have sought services, what motivated you to get help?
7. If you DID access mental health services, please indicate the reasons why you accessed mental health services (select all that apply):
  ∘ Cost effective
  ∘ Believe it would help
  ∘ Easy to access mental health services
  ∘ Felt like I had the time or energy
  ∘ Was interested in seeking services
  ∘ Believed I needed services
  ∘ Decline to Respond
  ∘ Other: _________________________
  **The following item appeared as a sliding scale (continuous variable)*
8. To what extent do you find each of the following to be a motivator to you using mental health services?
  ∘ Cost effective
  ∘ Believe it would help
  ∘ Easy to access mental health services
  ∘ Felt like I had the time or energy
  ∘ Was interested in seeking services
  ∘ Believed I needed services
  ∘ Important for my family
9a. How interested would you be in accessing [insert for each service in Q1 above] service for yourself?
  ∘ Not at all interested (1)
  ∘ A little interested (2)
  ∘ Neutral (3)
  ∘ Somewhat interested (4)
  ∘ Very interested (5)
9b. How interested would you be in accessing [insert for each service in Q1 above] service if it were integrated into the Neonatal Follow-up Program? *on a sliding scale*
  ∘ Not at all interested (1)
  ∘ A little interested (2)
  ∘ Neutral (3)
  ∘ Somewhat interested (4)
  ∘ Very interested (5)
10. What format would you prefer to access mental health services for yourself?
  ∘ In-person
  ∘ Virtually via telephone
  ∘ Virtually via video
